# Lipidomics, Microbiota, and Intestinal *Clostridioides difficile* Infection Outcome

**DOI:** 10.3390/ijms26178214

**Published:** 2025-08-24

**Authors:** Marija Branković, Marija Kraišnik, Dimitrije Zdravković, Nemanja Kraišnik, Filip Jelić, Novica Nikolić, Siniša Đurašević, Tomislav Tosti, Tijana Gmizić, Zoran Todorović

**Affiliations:** 1 University Hospital Medical Center Bežanijska kosa, 11000 Belgrade, Serbia; dukic.marija@bkosa.edu.rs (M.K.); nemanjakraisniknemanja@gmail.com (N.K.); filip.jelic@live.com (F.J.); nikolic.novica@bkosa.edu.rs (N.N.); gmizic.tijana@bkosa.edu.rs (T.G.); zoran.todorovic@med.bg.ac.rs (Z.T.); 2 Faculty of Medicine, University of Belgrade, 11000 Belgrade, Serbia; zdravkovic.dika@gmail.com; 3 Department for Comparative Physiology and Ecophysiology, Institute for Physiology and Biochemistry Ivan Đaja, Faculty of Biology, University of Belgrade, 11000 Belgrade, Serbia; sine@bio.bg.ac.rs; 4 Institute of Chemistry, Technology and Metallurgy, National Institute of the Republic of Serbia, University of Belgrade, 11000 Belgrade, Serbia; tosti@chem.bg.ac.rs

**Keywords:** lipidomics, microbiota, *C. difficile*, infection

## Abstract

It is mostly known which microorganisms make up the intestinal microbiota and what their role is in the digestive tract. Moreover, there is evidence about the influence of these microorganisms, especially bacteria, on the functioning of the human body in general. Intestinal microbiota is metabolically active and synthesizes numerous molecules that are an important part of biochemical processes in the human body, as well as signaling pathways. Some of these molecules are of lipid origin, which is why new knowledge in the field of lipidomics can help in their more precise identification. It is now clear that the lipid profile of the stool depends on the composition and metabolic activity of the microbiota. Therefore, under changed conditions, such as the existence of an infection, there are changes in the lipid profile of the stool. One of the epidemiologically most important and most studied infections of the digestive tract is *Clostridioides difficile* infection. This infection is recurrent in a large number of cases; it is related to resistance to antibiotics and their irrational use, and because of that, further research in this area would bring insight into possibly new knowledge that would help in more effective suppression of this infection.

## 1. Introduction

In recent years, the topics of various articles in the fields of medicine, genetics, chemistry, biochemistry, and microbiology represent achievements in the development of metabolomics, metagenomics, and lipidomics. This led to significant conclusions regarding the mentioned molecules, but also their connection with human health and disease. Through research, it became known that the molecules synthesized in the organism differ depending on whether the organism is in a state of physiological balance or imbalance, which made it possible to use these molecules as markers of certain diseases. The field of lipidomics began to develop about twenty years ago with the appearance of the term “lipidome” in one of the scientific journals [1]. The authors explained that this term encompasses a set of structurally different lipid molecules in a cell, organ, or organ system. Then the term “functional lipidomics” appeared, which primarily referred to cell membrane lipids, and its creators were Rildfors and Lindblom [2]. The further development of lipidomics led to the appearance of new terms and definitions, but the bottom line is that it has been proven that lipids play a crucial role in performing basic cellular processes and ensure the most important biochemical processes in the body. Lipidomics uses two lines of research: targeted lipidomics and untargeted lipidomics [3]. Targeted lipidomics aims to detect already known specific metabolites and determine what happens in the event of their alteration, while untargeted lipidomics is used to establish a hypothesis, that is, to identify unknown changes in both known and unknown molecules. Modern lipidomics enables precise characterization of the structure of lipids, determination of their concentration, detection of changes in the structure of lipids and the impact of these changes on the organism, and the application of all of the above in the development of new medical achievements, such as, for example, markers of certain diseases [4,5,6,7]. A special area of lipidomics is clinical lipidomics, which allows the knowledge gained from the study of lipids to be applied in a clinical setting [8,9]. Clinical lipidomics is of particular importance to medical professionals. It aims to apply the methods used in lipidomics to profile the types of lipids from biological samples that can be obtained in clinical settings. Most often, these are plasma and serum, but stool is also used. Lipids isolated in this way can represent markers that indicate the risk of a disease, complications, or death. In lipidomics, the most commonly used approach is based on mass spectrometry, which involves measuring the mass of molecules and classifying them based on this. Methods that rely on nuclear magnetic resonance are also in use. The latest techniques involve the creation of models using bioinformatics technologies and statistics [10,11,12]. The results obtained by any of these methods must undergo standardization in order to be understandable to clinicians, comparable, and applicable in clinical practice [13,14]. Regardless of the great progress and development of the technologies mentioned, the translation of the obtained results is not always possible, primarily due to the insufficient availability of clinical lipidomic assays.

Among the lipids that were first used as markers in clinical practice, very low-density lipoprotein, low-density lipoprotein, high-density lipoprotein, and total triglyceride concentration stood out [15]. A good example of a modern marker obtained by lipidomics methods and later verified in clinical studies is the use of ceramide levels for the assessment of cardiovascular risk [16]. Also, a model called CERT2, which includes ceramide and phosphatidylcholine levels, has been shown to be highly effective in predicting mortality from cardiovascular diseases [17]. This model was further improved when the highly sensitive troponin T was taken into account among the two lipids. Lipid biomarkers have also found their application in assessing the therapeutic effect of drugs; for example, the use of phosphatidylinositol and phosphatidylcholine in assessing the effect of pravastatin [18].

Lipids are complex macromolecules, and their diversity is supported by the fact that more than 40,000 different molecules are known [19]. Considering that they are products of the metabolic activity of cells, their research is also dealt with by metabolomics, which, it can be said, looks at metabolic processes more broadly than lipidomics, which is focused only on lipids [20]. The division of lipids into basic categories was determined by the LIPID MAPS consortium in 2005, and it has been changed several times so far. The basic categories according to this division are sphingolipids, saccharolipids, fatty acids, glycerophospholipids, glycerolipids, sterol lipids, and prenol lipids [21]. Initially, a special category was represented by polyketides, but they were subsequently excluded because their characteristics differ significantly from the other mentioned categories [21]. Each category is further classified into individual types depending on the polar group it possesses, and it can also be further classified into subtypes depending on the individual or structural characteristics of the molecule [21]. Some of them participate in the construction of cell and organelle membranes, and some of them are energy depots for carrying out cellular processes [22]. Small changes in the structure of lipids can change their activity in the cell [23,24].

The aim of this paper is to highlight the importance of knowledge of lipidomics and gut microbiota in the context of health and disease. This knowledge can, among other things, be used to more effectively diagnose, treat, and prevent the spread of *Clostridioides difficile* (*C. difficile*) infection as one of the most clinically prevalent and challenging infections and provide a greater chance of a positive outcome.

## 2. Intestinal Microbiota and Its Impact on Maintaining Health and the Onset of Disease

Hypothetically, it can be said that the intestinal microbiota represents an organism within the human organism. More than 1000 different species of bacteria make up the microbiota of a healthy person, but to a lesser extent there are also viruses, fungi, protozoa, and archaea [25]. The composition of the intestinal microbiota is variable, dynamic, and dependent on diet, lifestyle, and external factors, as well as the use of drugs [26,27]. It has been proven that the composition of the microbiota depends on the geographical area in which a certain population lives, considering the difference in the natural environment, diet, and external factors that are characteristic of a certain area [28]. In addition, in the process of aging, the organism undergoes changes, among other things, at the level of the microbiota, which means that its composition also depends on the age of the organism [29,30,31]. In elderly people, there is an increase in the number of bacteria that can be associated with the onset of disease, such as, for example, bacteria from the genera *Clostridioides* and *Bacteroides*, which also speaks in favor of a decrease in the number of bacteria that have a protective role [32,33]. The importance of the microbiota and its diversity is also reflected in the fact that the term gut microbiota genome is often used as a secondary human genome [34]. The bacteria that make up the microbiota are responsible for maintaining the state of equilibrium at the level of the intestine, and their metabolic activity can help the performance of various biochemical processes but also precipitate the onset of disease in certain situations [35]. They synthesize some of the necessary nutrients for the human body (for example, vitamin K and vitamin B), as well as proteins that can play a role in various signaling pathways [36]. They participate in the digestion process but also in the immune defense of the organism [37]. Intestinal dysbiosis is considered one of the key factors that contribute to the development of many chronic diseases, including inflammatory bowel diseases, diabetes mellitus, metabolic dysfunction-associated steatotic liver disease, other metabolic and autoimmune diseases, and colon carcinoma [38,39,40,41]. Also, studies have shown that intestinal dysbiosis can be associated with the onset and progression of obesity, but also with diseases characterized by metabolic disorders such as diabetes mellitus [42]. In 2018, Djurasevic et al. published an interesting study that aimed to examine the effect of virgin coconut oil on the composition of the microbiota, but also the glycemic profile, food and fluid intake, and weight gain of rats that had diabetes mellitus and a control group that did not have it [43]. Coconut oil was chosen because it is a source of short- and medium-chain fatty acids, which have been shown to have a beneficial effect on obesity. The results of this research showed that the effect of virgin coconut oil can be positive for the maintenance of normal intestinal microbiota, given that in the experimental group it was observed that there was an increase in the number of bacteria from the genera *Lactobacillus*, *Allobaculum*, and *Bifidobacterium*. Regarding the effect on glycemia, coconut oil caused a slight decrease in glycemic values, most likely because it contains lauric acid, which has insulinotropic properties. Also, rats fed virgin coconut oil gained weight faster, despite the fact that food and water intake was reduced, bearing in mind that it is a source of fatty acids in a high content and that it is an energy-efficient food. The impact of intestinal dysbiosis on the development of chronic diseases is evident if we know that the intestinal mucosal barrier is one of the first to protect the organism from the onset of disease. It aims to prevent the passage of pathogenic microorganisms, foreign antigens, and toxins further into the body, but also to ensure the constant exchange of nutrients, water, and other important molecules between the intestinal lumen and systemic circulation [44]. In situations where there is increased permeability of the intestinal barrier, the organism is susceptible to the onset of disease. The microbiota of a healthy person is mostly made up of bacteria from the genus *Bacteroidetes* (*Prevotella*, *Porphyromonas*) and *Firmicutes* (*Clostridium, Eubacteria*) [45]. The *Firmicutes/Bacteroidetes* ratio is a good indicator for assessing the existence of dysbiosis [46]. In addition to the mentioned species, there are also *Proteus*, *Actinobacteria* (*Bifidobacterium*), *Lactobacillus*, *Streptococcus*, *Escherichia coli*, and many others [47].

## 3. The Relationship Between Lipidomics and Intestinal Microbiota

**Figure 1:**
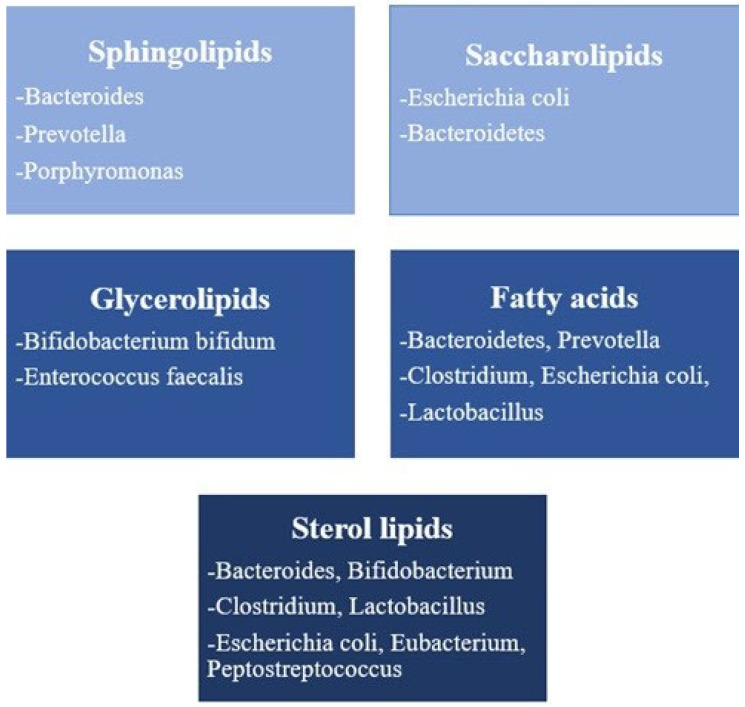
Bacteria and lipid classes in whose metabolism and synthesis they participate.

As previously mentioned, bacteria of intestinal microbiota are metabolically active and have a significant contribution to the synthesis of various molecules in the digestive tract. Their metabolic activity can significantly affect the condition of the host. For this reason, two terms are mentioned: gut microbiome-metabolome co-axis and microbial-host-lipid co-axis, which specifically concern metabolites synthesized by the bacterial flora of the intestine, among other lipids [48,49]. Despite the fact that microbiota produce a wide range of metabolically active molecules, only those that are lipophilic can pass the intestinal mucosal barrier and directly affect the host organism [50,51]. For this reason, the fecal lipidome can give us an answer to the question what the relationship between the host and the microbiota is [52]. This approach to the study of the microbiota and its, but also the lipidome of the host, has attracted a lot of attention recently. The limiting factor is that highly sensitive methods are needed to enable precise identification of lipid origin (whether it is of host or microbiota origin) but also determination of the exact amount in the stool, which is still not routinely possible [49]. Below, we will mention the classes of lipids, their characteristics, and whether members of the intestinal microbiota synthesize them (Figure 1).

### 3.1. Sphingolipids

**Figure 2:**
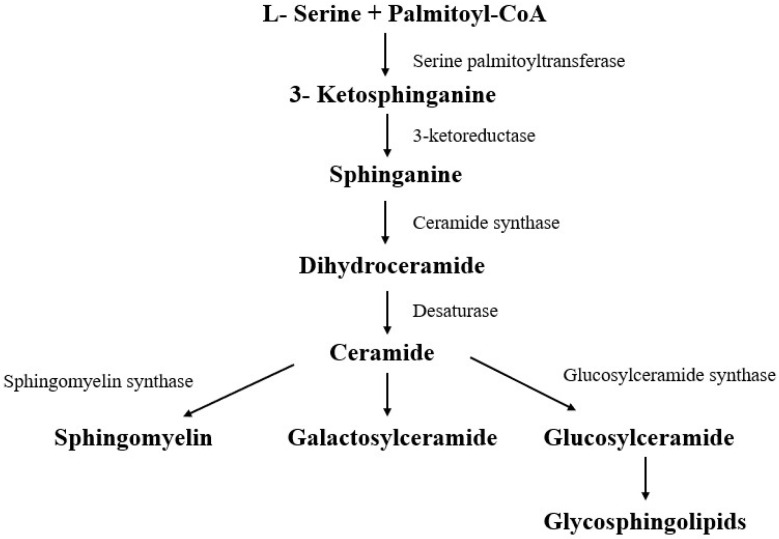
Biosynthesis of sphingolipids.

Sphingolipids are a category of lipids that have a sphingoid base and are synthesized in the endoplasmic reticulum of the cell from fatty acids and amino acids with the help of coenzyme A and most often serine palmitoyl transferase (since they are most often serine and palmitate) [53] (Figure 2).

Their roles are diverse; they represent one of the basic building blocks of cell membranes and participate in signal transduction. This category includes ceramides, sphingomyelins, and glycosphingolipids. Ceramides are the basis of sphingomyelins and glycosphingolipids, whose synthesis after the endoplasmic reticulum is further carried out in the Golgi apparatus. Also, ceramides have been the subject of many studies and have been found to be associated with the development of metabolic syndrome, cardiovascular, neurological, and nephrological diseases, but also to participate in the process of programmed cell death, which emphasizes their importance [54,55,56,57]. It is interesting that some bacteria can also synthesize sphingolipids [58,59]. The most important producers of sphingolipids in the intestines are *Bacteroides*, and besides them there are also *Prevotella* and *Porphyromonas*. Sphingolipids synthesized by *Bacteroides* have been shown to have an effect on ceramide levels in the host’s liver [60]. Also, *Bifidobacterium* does not synthesize sphingolipids but can use them from exogenously supplied nutrients [61]. The classes of sphingolipids, glycosphingolipids, and phosphosphingolipids are also synthesized by bacteria of the genus *Bacteroides.*

### 3.2. Saccharolipids

This is a category of lipids in which fatty acids are attached to a sugar backbone [62]. There are two main subclasses of saccharolipids, namely acylaminosugars and acyltrehaloses. The most widely known representative of saccharolipids is lipid A, which is the main component of the lipopolysaccharide of Gram-negative bacteria. After the breakdown of the bacterial membrane, lipid A is released, which has a harmful effect on the human body [63]. The best known are lipid A of *Escherichia coli* and the phylum *Bacteroidetes* [64,65,66].

### 3.3. Fatty Acids

These are carboxylic acids characterized by aliphatic chains, which in the cell can perform a function independently or participate in the construction of other lipids and molecules, which increases the spectrum of their roles [67]. They can be synthesized in the human body but can also be introduced exogenously. Fatty acids participate in the modulation of cell membrane stability. In relation to some characteristics, such as chain length, degree of saturation, and whether they are hydroxylated, they have different roles in the organism. According to the degree of saturation, they are divided into saturated and unsaturated fatty acids and, depending on the number of double bonds they have, into monounsaturated and polyunsaturated fatty acids [68]. According to the length of the chain, they are divided into short-, medium-, and long-chain fatty acids, and according to whether the organism can synthesize them or not into essential and non-essential [69]. Short-chain fatty acids have 1–6 carbon atoms, and the most important in the human body are propionates, acetate, and butyrate [70]. They are created in the digestive tract in the process of fiber fermentation by microbiota [71]. These acids, together with their receptors, regulate the metabolic activity of the cell. In addition, for example, butyrate has a role in the regulation of cell apoptosis and the control of cell proliferation [72,73]. Also, the role of short-chain fatty acids in the development of allergies, metabolic diseases, and autoimmune diseases is examined [74]. Medium-chain fatty acids have 6–12 carbon atoms, and long-chain fatty acids have 13–21 carbon atoms. Medium-chain fatty acids, as well as short ones, play a role in intracellular signaling; they are agonists of peroxisome proliferator-activated receptors and regulate cell proliferation and death [75,76]. Among long-chain polyunsaturated fatty acids, omega-3 and omega-6 are responsible for maintaining the fluidity of the cell membrane, have an anti-inflammatory effect, have a beneficial effect on repairing the function of endothelial cells of blood vessels, etc. [77]. Short-chain fatty acids are synthesized by bacteria from the genera *Bacteroides*, *Clostridioides*, and *Prevotella* from exogenously ingested dietary fiber [71]. Long-chain fatty acids are also synthesized by *Bacteroidetes* and *Prevotella* in a smaller amount, but also by some strains of *Escherichia coli* and *Lactobacillus* [78,79,80].

### 3.4. Glycerolipids

This is a class of lipids determined by the glycerol backbone to which the polar head and acyl chain are attached. The best-known representatives are glycerophospholipids, which are found in the lipid envelope of bacteria. In addition, some bacterial strains produce glycoglycerolipids, usually Gram-positive bacteria such as *Bifidobacterium bifidum* and *Enterococcus faecalis* [81].

### 3.5. Sterol Lipids

Sterol lipids are cyclic hydrocarbons that have hydrophobic characteristics. They are synthesized by both prokaryotic and eukaryotic cells. Bacteria have the ability to transform exogenously introduced sterols into various bioactive lipid molecules [82,83]. The most common sterol is cholesterol, which can be introduced into the body exogenously but is also primarily synthesized in the liver, where it is metabolized to primary bile acids. Then, at the level of the intestine, primary bile acids are transformed into secondary bile acids by microbiota. Secondary bile acids are deoxycholic acid, lithocholic acids, and ursodeoxycholate, as well as many others [84,85]. *Bacteroides*, *Bifidobacterium*, *Clostridium*, and *Lactobacillus* mainly participate in the transformation of primary into secondary bile acids, similarly to *Escherichia*, *Eubacterium*, and *Peptostreptococcus* [86,87]. Under physiological conditions, secondary bile acids make up only 10% of the total amount of bile acids, which is important to know due to pathological conditions in which this percentage increases, which means that the influence of secondary bile acids on the host’s metabolism also increases [51,88]. Considering the above, it can be concluded that the intestinal microbiota influences the level of cholesterol in the host organism [89].

## 4. Intestinal Microbiota and Its Role in Drug Biotransformation—Is There a Connection with Lipidomics?

Previous research has shown that the metabolic activity of intestinal microbiota in the biotransformation of drugs can also affect lipidomics in the gastrointestinal tract. This activity is diverse, but it is known that the most common reactions include hydrolytic reactions and reductive metabolism. In addition, decarboxylation, deamination, dealkylation, dihydroxylation, and dehalogenation are also mentioned. Microbiota bacteria possess enzymes that participate in the aforementioned reactions and thus participate in drug metabolism, for example, beta-glucuronidases and sulfatases [90,91,92]. With their help, they metabolize drugs into active metabolites. The products of these metabolic reactions are different and can still significantly affect the host organism. Metabolites formed in this way can have an impact on the host’s lipid metabolism genes; for example, reduced expression of fasting-induced adipose factor leads to increased storage of fatty acids in adipose tissue and liver, as well as an inhibitory effect on intestinal lipoprotein lipase, and increased expression of the gene for the synthesis and release of peptide YY leads to a slowdown in intestinal motility [93,94,95]. In addition, the metabolic products produced in the previously mentioned way can directly affect the levels of phosphatidylcholine and cholesterol in the intestines [96,97]. The effect of metabolites, created by the activity of microbiota enzymes, on the metabolic pathways that regulate the synthesis of lipid metabolism enzymes, for example, Toll-like receptors, was also shown [98]. *Bacteroides* lipopolysaccharide can act as an agonist for Toll-like receptors type 2 and 4 and thus trigger an inflammatory response. Other metabolites produced by the biotransformation of drugs by microbiota enzymes can contribute to the development of inflammation and affect the profiles of phospholipids and sphingolipids in the intestinal epithelium [98]. Knowledge of these interactions is extremely important considering that today the use of drugs for various indications is expanding. For example, nonsteroidal anti-inflammatory drugs are widely used to achieve anti-inflammatory and analgesic effects. Bacterial beta-glucuronidases can lead to the deconjugation of conjugated nonsteroidal anti-inflammatory drugs with a carboxyl group (e.g., diclofenac), releasing an aglycon that disrupts intestinal integrity, increases lipid resorption, and affects the intestinal lipidome [99]. Also, the effectiveness of drugs in the interaction with the microbiota can be reduced because this interaction can change the lipid milieu (e.g., phosphatidylcholine levels) and thus the solubility of the drugs [100]. Many substances that are used in the foods that we use every day can also be a source of metabolites that will be created under the influence of microbiota and increase the risk of various diseases. For example, azo compounds that are used as food additives but also in the pharmaceutical, paint, textile, and printing industries, can be activated under the influence of intestinal bacteria, which leads to the release of metabolites that block the metabolism of polyunsaturated fatty acids and reduce the content of arachidonic acid in the intestines, and the consequences are numerous—inflammation, metabolic disorders, and cancer [101].

## 5. *Clostridioides difficile*—Today’s Epidemiological Problem in Hospital Conditions

*C. difficile* is a Gram-positive, anaerobic, sporogenous bacterium that is a common cause of antibiotic-associated diarrhea. Bacteria from this genus are normally found in a certain number in the human intestinal microbiota, but by changing the conditions, they can become dominant and cause infection [87,102]. This bacterium was first isolated in 1935 from the feces of a newborn, and for many years after, it was not considered dangerous for human health [103,104]. Pseudomembranous colitis, which we now know is caused by *C. difficile*, was attributed to *Staphylococcus aureus*. This opinion persisted until 1978, when Bartlett et al. published a paper mentioning the possibility of toxin production by *C. difficile*, which could be an explanation for pseudomembranous colitis [105]. *C. difficile* is transmitted by the fecal–oral route and can cause a mild, moderate, or severe clinical picture of the infection [106]. It is a sporogenous bacillus whose spores are resistant to most preparations used for disinfection, and this is the reason why they survive even in an acidic stomach environment [107]. For example, they are resistant to ultraviolet (UV) radiation, oxygen, and heat [108]. The mere presence of bacteria or its toxins does not mean infection if there are no symptoms. In that case, no treatment is necessary. However, if the presence of bacteria and/or its toxins is confirmed, and symptoms such as diarrhea, abdominal pain, and fever are present, therapy is required, as that indicates an infection [109,110]. The clinical picture of infection is most often caused by the production of two toxins, which are called toxin A and toxin B. Strains of bacteria that produce toxins are more invasive than those that do not produce them [111]. Also, *C. difficile* has the ability to synthesize other molecules that enable its survival and reproduction at the level of the digestive tract, such as adherence factors, proteolytic enzymes, and molecules that help it form a biofilm [112,113]. The severity of the clinical presentation of an infection depends on both bacterium-related and host-related factors. Factors related to bacteria are the ability to produce toxins, the presence of the aforementioned molecules that enable colonization, and the number of colonies. Factors related to the host are its defense capacity (immunity), age, existence of other chronic diseases, previous hospitalization, whether antibiotics are used and which ones, whether proton pump inhibitors or immunosuppressants are used, etc. [106,114,115,116]. In cases where the infection is severe or the patient is immunocompromised, an unfavorable clinical course may occur, which would include a severe form of pseudomembranous colitis with the risk of colon perforation, but also septic shock [114].

**Figure 3:**
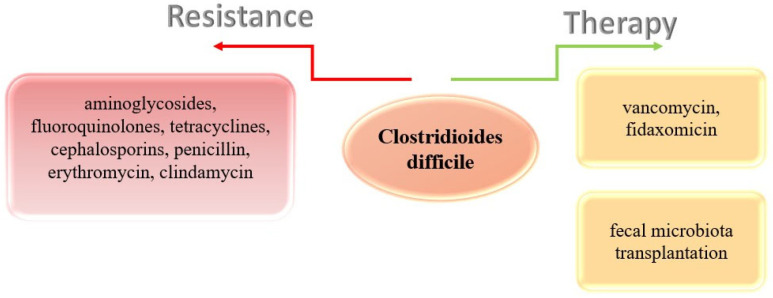
*Clostridioides difficile* resistance to antibiotics and available therapy.

Nowadays, *C. difficile* is the most common intrahospital infection, the cause of which, in addition to the mentioned factors, is also the irrational use of antibiotics. The greatest risk for infection is the use of broad-spectrum antibiotics, which is also the reason for the high resistance of this bacterium to antibiotic therapy [117,118,119]. So far, resistance of *C. difficile* to aminoglycosides, fluoroquinolones, tetracyclines, cephalosporins, and penicillin, but also representatives of macrolides (erythromycin) and lincosamine (clindamycin), has been proven (Figure 3) [117].

There are also data on resistance to metronidazole, but additional studies are needed [120,121]. Vancomycin and fidaxomicin are used to treat this infection, and nowadays, more often, to treat fecal microbiota transplantation [122,123]. This infection represents an epidemiological challenge in hospitals around the world and multiplies the cost of treatment and the duration of hospitalization of patients [124]. According to some data, about 124,000 cases of *C. difficile* infection are reported every year in the European Union, and this number is increasing year by year despite the increased awareness of this infection [107]. The most common epidemics occur in hospitals, nursing homes, rehabilitation clinics, and environments where hygienic conditions are poor. The infection also spreads thanks to the existence of asymptomatic carriers [125]. Thanks to numerous studies that have been conducted on this topic, it has been established that there are strains of bacteria that are currently particularly resistant and invasive, such as polymerase chain reaction (PCR) ribotypes 027 [108]. Due to all of the above, it can be concluded that, considering that the therapeutic options for treating this persistent infection are few, adequate measures to prevent its spread should be implemented, as well as raising awareness about it in risky environments.

## 6. The Importance of Knowing Lipidomics and Microbiota for the Outcome of *Clostridioides difficile* Infection

**Figure 4:**
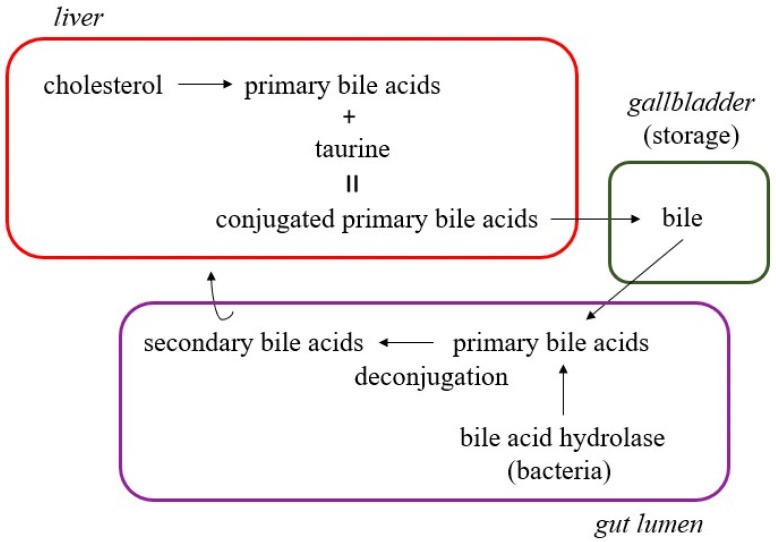
Transformation of primary to secondary bile acids by gut microbiota enzymes.

We have already emphasized the importance of the relationship between lipidomics and the intestinal microbiota. If we bear in mind that scientific methods and technologies are almost constantly advancing today, the question can be asked whether knowing the lipid profile of the stool will give us the answer to the so far unanswered question—how to prevent and more effectively treat *C. difficile* infection? Also, previous knowledge about the composition of the microbiota as well as its synthetic possibilities has led to significant advances in the innovative therapy of *C. difficile* infection. One of these is certainly the use of fecal microbiota transplantation for the treatment of recurrent *C. difficile* infection [126,127]. Recurrent *C. difficile* infection occurs in approximately 25% of patients and is defined as the recurrence of symptoms of infection with a positive test within eight weeks of completing therapy [128,129]. Fecal microbiota transplantation involves the use of stool from a healthy donor to re-establish the dominance of beneficial bacteria over *C. difficile* in an infected person [128]. The exact mechanism by which host microbiota, or those colonized by fecal microbiota transplantation, suppress *C. difficile* colonization is still unclear. Several hypotheses exist, and they link knowledge from lipidomics, primarily bile acid metabolism, and the gut microbiota. The microbiota primarily participates in the transformation of primary to secondary bile acids [130,131] (Figure 4).

Some bile acids, such as chenodeoxycholate, have been shown to inhibit the germination of *C. difficile* spores [132,133,134]. Secondary bile acids produced by the microbiota can also inhibit the growth of the vegetative form and reduce toxin activity [134,135]. Cholate, chenodeoxycholate, lithocholate, and deoxycholate have been shown to attenuate the effects of toxin B by binding to it [136]. All of these may increase the chances of successful fecal microbiota transplantation, especially in recurrent infections, as well as the chances of a positive outcome of infection [137,138]. Bacteria from the phylum *Bacteroidota*, *Actinomycetota*, and some others synthesize and release bile acid hydrolases that deconjugate bile acids. This is intended to inhibit the growth of the vegetative form of *C. difficile* [139,140]. Hydroxysteroid dehydrogenases are enzymes that certain members of the microbiota use in some cases for deconjugation of bile acids [141]. This process produces epimers of lithocholate that inhibit the growth of *C. difficile* and reduce the expression of its toxins [136,142].

In addition to the aforementioned enzymes involved in lipid metabolism, the presence of the bile-acid-induced (bai) operon has been identified in the intestine of healthy centenarians [143]. This operon contains six genes responsible for the dehydroxylation process at the 7α position. Removal of the hydroxyl group at this site converts primary to secondary bile acids. Secondary bile acids produced in this way (deoxycholate, isodeoxycholate, lithocholate, isolithocholate, and hyodeoxycholate) inhibit the germination of spores, the growth of the vegetative form of bacteria, and the activity of toxins [144]. Also, it is known that chenodeoxycholic acid is a farnesoid X receptor agonist. *C. difficile* produces tyrosicholic and phenylalanocholic acid, which are agonists of the same receptor. In this way, through farnesoid X receptor, *C. difficile* upregulates the release of enterohepatic hormone secreted in the ileum named fibroblast growth factor 19, which influences the downstream regulation of the enzyme CYP7A1 that converts cholesterol into bile acids, resulting in inhibition of primary bile acid synthesis [145,146]. In addition, farnesoid X receptor signaling has an impact on immunity and cell differentiation, which means that in conditions where there is a *C. difficile* infection, this relationship can also be disturbed. In addition, we mentioned earlier that in physiological conditions, secondary bile acids make up only 10% of total bile acids, which means that in conditions of infection with *C. difficile*, this percentage increases. Therefore, the influence of secondary bile acids on the host’s organism also increases. Secondary bile acids in elevated concentrations can lead to increased permeability of the intestinal mucosal barrier and increased risk of inflammation but also to colorectal cancer [147]. Also, Kenny et al. identified an enzyme by which *Eubacterium coprostanoligenes* affects cholesterol metabolism—it converts it into cholestenones [148]. Bacteria from the genus to which *C. difficile* belongs have similar enzymes. The lipid profile of stool changes in the following ways during *C. difficile* infection. With the latest advances in lipidomics and determination of the lipid profile of stool, we are closer to finding markers that could indicate the risk of more serious forms of infection and its complications. Timely action in these cases, as well as the selection of adequate therapy, would reduce morbidity and mortality of *C. difficile* infections. Determining the lipid profile of the stool can be a step towards a better understanding and control of this infection.

## 7. Conclusions

*C. difficile* is one of the infections whose control still represents a global challenge for medical experts. Disruption of healthy intestinal microbiota and the use of antibiotics are the main predisposing factors for the development of infection. The intestinal microbiota, with its complex interactions, affects metabolic activity at the intestinal level, including lipid metabolism. Changes in lipid composition can positively or negatively affect the risk of colonization by *C. difficile*. Such changes affect the lipid profile of the stool, which can help us identify risk factors for the development of more severe forms of infection, as well as recurrent infection. In addition, knowledge of changes in lipid metabolism at the level of the intestine, both of host origin and microbiota, can ensure greater effectiveness of new therapeutic methods such as fecal microbiota transplantation. All of the above increases the chance of faster recovery from *C. difficile* infection, as well as its more effective suppression, but also reduces the costs of treating the infection and its complications. Despite all the aforementioned facts, more research is needed in this area in order to be closer to understanding the importance of the role of lipids in overcoming *C. difficile* infection, but we can safely say that science is on the right track. 

## Data Availability

Not applicable.

## Abbreviation

The following abbreviation is used in this manuscript:

*C. difficile*	*Clostridioides difficile*
